# Hospital Agios Dimitrios: The General Public Hospital of Thessaloniki

**DOI:** 10.7759/cureus.25685

**Published:** 2022-06-06

**Authors:** Spyros N Michaleas, Gregory Tsoucalas, Markos Sgantzos, Georgios Androutsos, Marianna Karamanou

**Affiliations:** 1 Department of History of Medicine and Medical Ethics, National and Kapodistrian University of Athens, Athens, GRC; 2 Department of History of Medicine and Medical Deontology, University of Crete, Heraklion, GRC; 3 Department of Anatomy, University of Thessaly, Larissa, GRC

**Keywords:** refugees, medical care, ministry of health, eleni zografou, history of medicine

## Abstract

The purpose of this article is to highlight the history of Hospital Agios Dimitrios, the General Hospital of Thessaloniki. During the early 20th century, many refugees settled in the Greek city of Thessaloniki. To address the growing public health needs of the city’s inhabitants, the Greek government established a health agency to offer medical care and respond to infectious disease outbreaks and epidemics. This initiative resulted in the construction or renovation of various hospitals. The Hospital Agios Dimitrios in Thessaloniki was completed in 1903. Its innovative architecture includes kiosks and wards designed to provide better ventilation and prevent the transmission of infectious diseases.

## Introduction and background

This article highlights the history of Hospital Agios Dimitrios, the General Hospital of Thessaloniki. Thessaloniki has great historical and strategic importance in Greece and is the capital of the geographic region of Macedonia. It has been home to thousands of refugees who fled to Greece during conflicts in the nineteenth and twentieth centuries [1]. To address the public health concerns and welfare of the city’s inhabitants, the government began constructing hospitals in the 1850s. Hospital Agios Dimitrios was finally completed around 1903 with a capacity of 100 beds. Still one of the most important hospitals in the city, Hospital Agios Dimitrios is named for the patron saint of Thessaloniki, Saint Demetrius. The innovative design of the building includes wards and kiosks with improved ventilation to reduce the transmission of infectious diseases, such as malaria, dysentery, and tuberculosis [2]. Demetrius (280-306) was an ecclesiastical figure and martyr who martyred under Diocletian (244-311) and became the patron and protector of the region. He was also a Roman officer who became a Christian and taught the new religion [3].

## Review

History of Thessaloniki

The history of Thessaloniki dates back to 316 BC, during the reign of Κing Cassander of Macedonia (350-297 BC), who was appointed regent by King Philip III (359-317 BC). After the death of Philip III, Cassander settled in Macedonia and married princess Thessalonike (352-295 BC), daughter of King Philip II (382 BC-336 BC) and half-sister of Alexander the Great (356-323 BC), with the aim to become king of Macedonia [4,5]. Κing Cassander honored his wife by naming the city of Thessaloniki after her [5].

**Figure 1:**
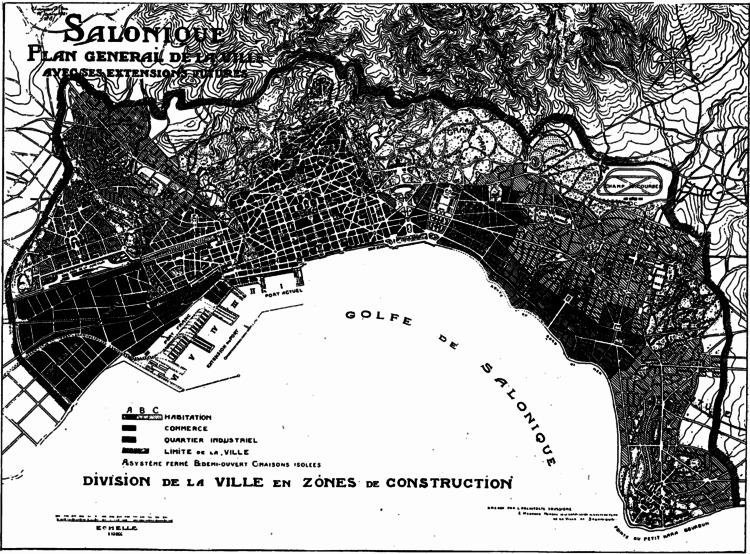
The city of Thessaloniki (1918) Yerolympos, A: Urban transformation in the Balkans (1820-1920): aspects of Balkan town planning and the remaking of Thessaloniki. University Studio Press, Thessaloniki; 1998.

Thessaloniki became a junction connecting the Adriatic Sea with the Hellespont and Asia Minor, allowing it to quickly develop into one of the most wealthy and populous cities in the region (Figure 1). In 168 BC, Lucius Aemilius Paullus (229-160 BC) defeated Perseus (212-166 BC), the last king of Macedonia, and captured Thessaloniki. By 146 BC, Thessaloniki was considered the center of the Provincia Macedoniae (Province of Macedonia) of the Roman Empire [6]. Christianity was later introduced to the province by the Apostle Paul (c. 5/15-66/68 AD), around 50-57 AD [7].

During the Byzantine era in 330 AD, around the time that Constantine the Great (272-337 AD) founded Constantinople, cities such as Thessaloniki began strengthening their commercial, military, and economic influence to maintain their roles as important crossroads between the West and the East [5,8]. After centuries of Greek control, Thessaloniki fell to the Ottomans on March 29, 1430, after slaughtering its inhabitants and converting many churches into mosques [8]. The city remained part of the Ottoman Empire until the Greek War of Independence (1821-1830). By that time, Thessaloniki had declined commercially due to war, taxation, the plague of 1837, and the fire of 1839 [9]. During the First Balkan War (1912-1913), the Greeks finally took back control of Thessaloniki and liberated the city on October 26, 1912.

In the aftermath of the Balkan Wars, Thessaloniki received a large wave of refugees from Serbia, Russia, the Caucasus, Bulgaria, and Anatolia [1]. Thousands settled in the city and its surrounding areas, such as Toumba, Lebet, and Kalamaria. The overcrowding raised fears of epidemics, leading the state to impose a quarantine. With no space to house the quarantined, the government proceeded to requisition private housing and public spaces [6].

Thessaloniki again served as an important strategic location during World War I (1914-1918), with British, French, and Serbian troops relying on its harbor for shipping supplies [10]. After Russia and the Central Powers signed the Treaty of Brest-Litovsk on March 3, 1918, large numbers of Greek refugees began arriving in Thessaloniki (1918-1920), but many died because of a mandatory six-month quarantine imposed by the government, a high fatality rate ensued. The Ministry of Health thus established a special mission in May 1919 to provide care to these refugees [11]. Many suffered from dysentery, malaria, tuberculosis, and typhoid [12], but the city had only about 100 beds, and only 32 of its 240 communities were served by a trained physician [13].

Newcomers who settled in Thessaloniki were attracted to its multiculturalism, housing, and employment opportunities. Several Muslim neighborhoods comprising approximately 9,000 properties had been deserted after the Treaty of Lausanne on July 24, 1923. During the first months after the Asia Minor Catastrophe (August 1922 - May 1923) about 117,000 refugees from Thrace and Asia Minor arrived in the city [1]. Refugees sought work and lodging [2]. They slept in abandoned buildings and homes, leading the government to convert temples, schools, and mosques into housing [14]. By 1932, 36 refugee settlements had been built, including Toumba, Kallithea, Saranta Ekklisies, Agia Fotini, Triandria, Kalamaria, and Eptalofos. However, most lacked even basic infrastructures, such as water, plumbing, and electricity [12,14].

Other hospitals in the region

As early as the 17th century, the Turkish chronographer and traveler Evliya Çelebi (1611-1682) mentioned the existence of a Greek Hospital in the city of Thessaloniki [15]. By the 19th century, Thessaloniki had several health centers, including the Community Asylum (circa 1847), the French Hospital of Brothers of Mercy (1893), Theagenio (1863), and the Hospital Regina Margherita (1894). Despite the abundance of health facilities, the region lacked an overall public health agency to coordinate the care of its citizens [16]. Thus, in 1917, Greek statesman and leader of the Greek national liberation movement, Eleftherios Venizelos (1864-1936), established the Ministry of Health [17].

After the turn of the 20th century, several more hospitals were built in Greece, including the Jewish Hirsch Hospital (1908), Hagia Sophia Russian Hospital (1910), Hospital for Special Diseases or "Smallpox House" (1912), Hospital for Venereal and Skin Diseases (1917), and Sanatorium of Asvestochori (1920). Another notable facility, Red Cross Hospital (1919), is still operating today as the Georgios Gennimatas General Hospital of Thessaloniki [16,18].

Agios Dimitrios Hospital

**Figure 2:**
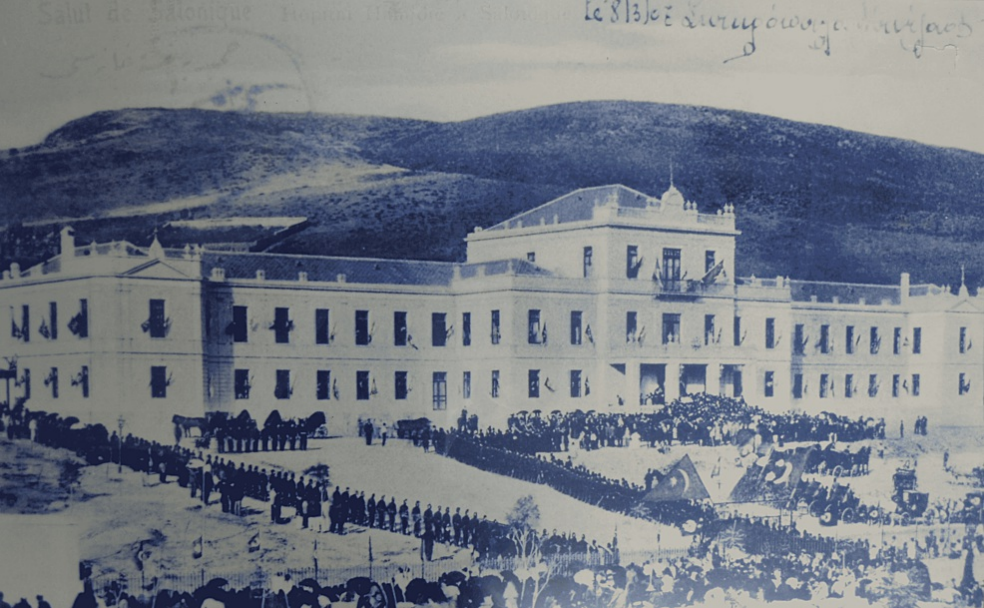
The inauguration of the hospital with the presence of the army Source: Archives of Agios Dimitrios Hospital

Agios Dimitrios Hospital was built in an area that the Greek community used for the burial of the dead [16]. Construction began between 1902 and 1903. On August 19, 1904, at the behest of Thessaloniki Mayor Hulusi Bey, the Hospital for Destitute Foreigners (*Gureba Hastahanesi*) was inaugurated [19] (Figure 2). The building, which sat on 54 acres (54,063.46 m^2^), was later converted to a Municipal Hospital (*Belediye*) and renamed Hamidiye Belediyesi in honor of Sultan Abdul Hamid II (1876-1909) [19]. According to the 1904 census, the city had about 129,796 inhabitants [20].

**Figure 3:**
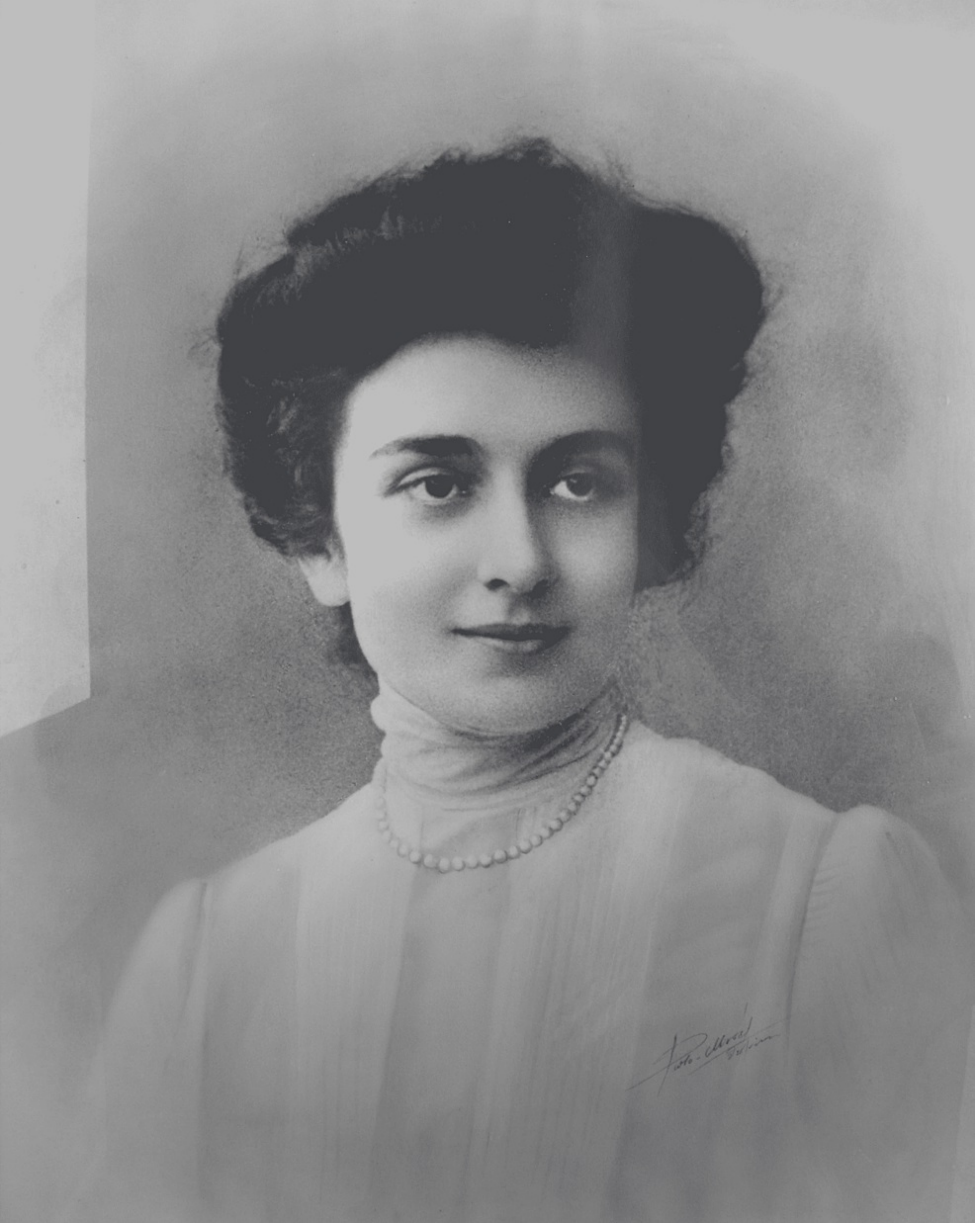
Eleni Zografou Source: Archives of Agios Dimitrios Hospital

**Figure 4:**
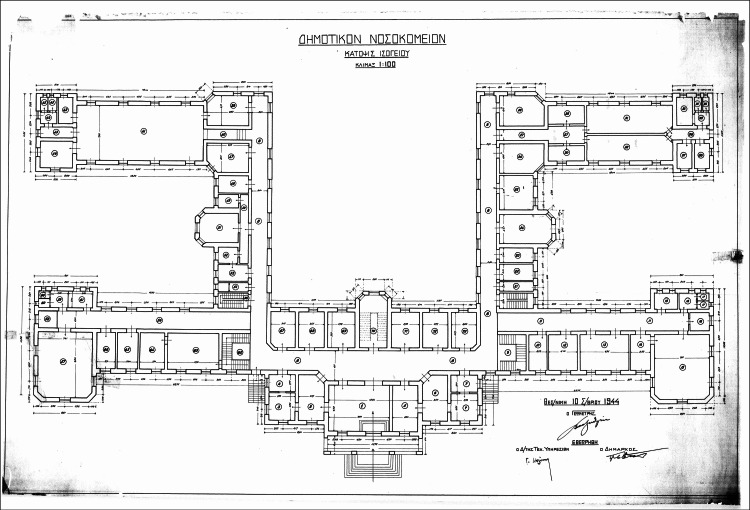
Ground floor plan of the main building of the hospital (1944) Source: Archives of Agios Dimitrios Hospital

**Figure 5:**
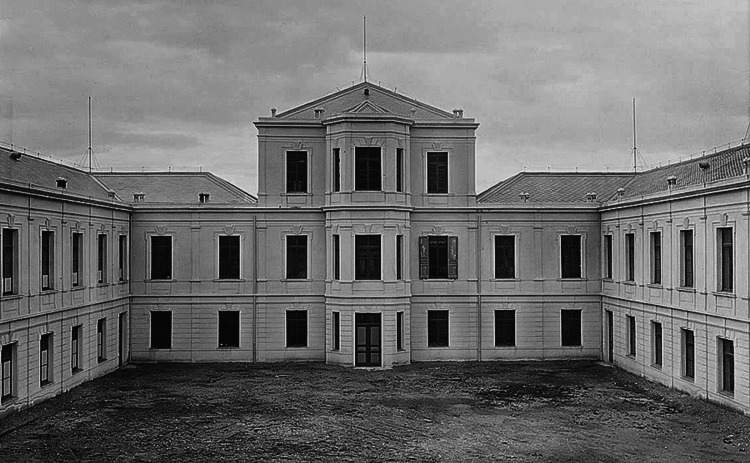
The main building of the hospital Source: Archives of Agios Dimitrios Hospital

In 1929, the daughter of famous Greek architect Lysandros Kaftantzoglou, Eleni Zografou (1872-1937) (Figure 3), left half of her property (45,946.46 m2) to the Municipal Hospital of Thessaloniki [2]. The hospital honored her by giving her name to the Internal Medicine Clinic. The building was located in the northern part of the plot, along with buildings hosting the governor of Thessaloniki and the Pasha Gardens, a one-acre green space of unknown origins. It was built in a "Π" shape with the facade facing the sea (Figure 4). The hospital building contained wards and kiosks designed to offer better ventilation and reduce the transmission of infectious diseases (Figure 5). The southern part of the property housed an anti-rabies clinic, and the northeast part an anti-tuberculosis clinic (demolished in 1955) [2].

The first Director of the Surgical Department, Panagiotis Oikonomou, had offices on the first floor of the main two-story building, along with the doctor on duty, outpatient room, pharmacy (which employed four pharmacists and three assistants), electric room, massage room, disinfectant room, baths and cold showers, laboratory, restaurant, kitchen, and laundry. The second floor included a men's operating room with 25 beds, two women's operating rooms with five beds each, two men's ward rooms with 15 beds each, a women's ward with 20 beds, a tuberculosis room with five beds, two ophthalmology wards with five beds each, and doctors' offices and dormitories. An adjoining room housed the furnaces for sterilization of tools, cotton, and bandages, as well as machines for sterilizing the water and heating patients' clothes [19].

After the liberation of the city in 1912, the Hellenic Municipal Hospital of Thessaloniki was managed by the *Adelfato *(Brotherhood), an administrative council appointed by the Central Administration of Macedonia. *Adelfato *was chaired by the mayor, and its members were elected by the Municipal Council. Over the next several years, a radiology laboratory, a department of pathology and ophthalmology, and a tuberculosis clinic were established, and the number of beds increased to 200 [19]. Additional clinics followed, including surgical, orthopedic, gynecological-obstetric, otolaryngological, pediatric, and urological clinics. In October 1936, a large fire broke out in the hospital, resulting in severe damage [2,16].

During World War II (1939-1945), the hospital supported the Greek army, which used much of the main hospital building as medical and storage facilities [2]. The hospital continued to face financial problems during the occupation of Greece by the Axis Powers (1941-1944), and the military hospital ceased to operate [2]. Agios Dimitrios Hospital continued to treat wounded Greek soldiers in conjunction with the Red Cross and the gendarmerie. In October 1943, the first University Clinics began operating, including psychiatric neurology, surgery, pathology, ophthalmology, and otolaryngology specialties [2].

On January 20, 1949, the hospital was bombed by the destroyer *Adrias *after a poorly regulated firing. The shell destroyed part of the patients' wards and seriously injured doctors, nurses, and patients. All the windows of the hospital were broken, and the machines of the chemistry were destroyed [2].

In 1971, the hospital became the property of the state as a Legal Entity under Public Law, and it was officially renamed the Hospital Agios Dimitrios in honor of the patron and protector of the city, Saint Demetrius [2,16,19]. It is still managed by the Administrative Council, which oversees the administrative, financial, medical, nursing, and pharmaceutical departments. Since 1975, pathology, surgery, ophthalmology, urology, otolaryngology, neurology, emergency, surgery, and microbiology clinics have been added, as well as a treatment center for incarcerated individuals [2].

Since 1980, the following departments have been added: audiometry, electroencephalography, nystagmography, neurochemistry, hematology and blood banking, and neuroradiology. The kitchen building was also expanded to increase the size of the restaurant and food storage. A prefabricated building was delivered to the hospital to accommodate more outpatient treatment and administrative services [2]. In 1982, physiotherapy, otolaryngology, and endoscopy services were added, followed shortly by cardiology, nephrology, hypertension, gastroenterology, Parkinson's disease, and the city's only 24-hour emergency dental care [2]. By 1989, orthopedic, gynecological, and intensive care units were added, along with pathology, hematology, endoscopy, ultrasound, angiography, and interventional radiology outpatient labs [2].

In the 1990s, doctors' locker rooms and gynecology offices were added to the ground floor, and a pathology clinic, kitchen, and new lavatories to the second floor. A new building was constructed to house the outpatient clinics, administration offices, library, and amphitheater. Outpatient clinics for autoimmune diseases, diabetes, lipidemia, headache, rheumatology, and fatigue testing also were added. Since the 2000s, social work, community outreach, breast health and mammography, sleep and apnea, orthopedics, pulmonary health, and diode laser services have been established [2]. Today, the Hospital Agios Dimitrios offers a wide variety of preventive and curative treatments for all ages, from vaccines to dentistry to nutrition [2,16].

## Conclusions

Thessaloniki has been a strategically important city from the time of Philip II until World War II. Under the rule of its many conquerors, the city also has been a major refugee settlement and multicultural center in the region. Overcrowding in the city has led to many disease outbreaks, such as malaria, tuberculosis, dysentery, and typhoid. Yet, until 1917, Thessaloniki had no organized public health policy. Soon after Eleftherios Venizelos established the Ministry of Health, the construction of hospital structures began. Hospital Agios Dimitrios in Thessaloniki, which was first erected in the 1850s and completed around 1902, was originally intended as a burial ground. The building has undergone numerous renovations in the last century and now serves as one of the most important public hospitals in the city.
